# Effects of vitamin D_**3**_ supplementation on cardiac, muscle, and immune responses after a marathon race: a single-blind, placebo-controlled trial

**DOI:** 10.1080/15502783.2026.2657316

**Published:** 2026-04-12

**Authors:** Pei‐Wei Weng, Josephine Diony Nanda, Yu-Hsiu Chien, Chiou-Feng Lin, Shih-Chung Cheng, Ming-Ta Yang

**Affiliations:** a Department of Orthopaedics, School of Medicine, College of Medicine, Taipei Medical University, Taipei, Taiwan; b International Ph.D. Program in Biomedical Engineering, College of Biomedical Engineering, Taipei Medical University, Taipei, Taiwan; c Department of Microbiology and Immunology, School of Medicine, College of Medicine, Taipei Medical University, Taipei, Taiwan; d Department of Parasitology, Faculty of Medicine, Public Health and Nursing, Universitas Gadjah Mada, Yogyakarta, Indonesia; e Graduate Institute of Athletics and Coaching Science, College of Athletics, National Taiwan Sport University, Taoyuan, Taiwan; f Graduate Institute of Medical Sciences, College of Medicine, Taipei Medical University, Taipei, Taiwan; g Center for General Education, Taipei Medical University, Taipei, Taiwan; h Clinical Research Center, Taipei Medical University Hospital, Taipei, Taiwan

**Keywords:** Catalase, cardiac troponin I, creatine kinase, lactate dehydrogenase, protein carbonylation, superoxide dismutase

## Abstract

**Background:**

Strenuous endurance exercise imposes substantial physiological stress on the cardiovascular system and has been associated with transient elevations in cardiac biomarkers. Vitamin D₃ has been suggested to influence oxidative stress and immune responses. In this study, we investigated the effects of vitamin D₃ supplementation on biomarkers of cardiac, muscle, and immune responses following a marathon race.

**Methods:**

Twenty-one amateur runners were allocated to either a vitamin D₃ supplementation group (receiving vitamin D₃ for 8 weeks) or a placebo group. All participants completed an official full marathon (42.195 km). Blood biomarkers were measured from 24 h before to 24 h after the race.

**Results:**

Post-race increases in markers of muscle damage and cardiac stress were observed. Vitamin D₃ supplementation was associated with attenuated elevations in selected biomarkers demonstrating significant interaction effects. Compared with the placebo group, the vitamin D₃ group demonstrated attenuation of protein carbonyls (PC), the only oxidative stress marker showing a significant Group × Time interaction. No significant interaction effects were observed for thiobarbituric acid reactive substances (TBARS) or antioxidant enzymes. Both groups showed post-race increases in white blood cell counts, particularly neutrophils, whereas lymphocyte counts significantly decreased at 0.5 h and 2 h post-race. Immunoprofiling revealed time-dependent alterations in selected immune cell subsets, although no significant interaction effects were detected. Descriptive differences in recovery patterns were observed between groups, and exploratory correlation analyses suggested time-specific associations between immune cell subsets and biochemical markers during recovery.

**Conclusion:**

Vitamin D₃ supplementation may attenuate PC responses and was associated with lower creatine kinase (CK) and creatine kinase-MB (CK-MB) levels at 24 h post-race following marathon running. Immune alterations were time-dependent, with descriptive differences in recovery patterns between groups.

## 1. Introduction

Marathon running is a globally popular endurance event, with approximately 1.2 million runners participating in major city marathons over the past half-century [1]. However, marathons are associated with a risk of sudden cardiac death: the incidence of sudden-onset cardiac death among participants ranges from 0.6 to 1.9 per 100,000 marathon runners [2]. Studies have reported diminished health benefits from prolonged, high-intensity exercise [3,4], with research indicating post-marathon increases in inflammation and oxidative stress that may be attributable to elevated blood neutrophil and monocyte counts and cytokine levels [5,6]. Prolonged endurance exercise can induce elevations in circulating cardiac biomarkers, including creatine kinase-MB and cardiac troponin I [7–9]. While these increases are often transient, considerable inter-individual variability exists in their magnitude and recovery kinetics [8–11]. The potential modulatory role of nutritional strategies in exercise-induced physiological stress responses warrants further investigation [12].

Vitamin D is a fat-soluble vitamin involved in immune regulation, inflammation suppression, modulation of cell proliferation and differentiation, and neuromuscular function [13–16]. A previous placebo-controlled intervention study in humans performing strenuous endurance exercise reported that vitamin D₃ supplementation attenuated markers of exercise-induced oxidative stress, such as protein carbonyls and TBARS [17]. Another study involving nonathlete men who performed exhaustive aerobic exercise reported that vitamin D supplementation increased antioxidant enzyme levels [18]. Collectively, these findings suggest that adequate vitamin D status may contribute to mitigating exercise-induced oxidative stress. Vitamin D deficiency or insufficiency is a common problem among athletes [19]. Therefore, investigating the applications of vitamin D in sports medicine is imperative. Studies on vitamin D supplementation in athletes have primarily explored its effects on exercise performance, muscle damage, and inflammation [12,19–21]. However, data regarding the direct effect of vitamin D on athletes’ health after marathons are limited.

Athletes participating in prolonged endurance training or competitions may experience transient increases in circulating cardiac biomarkers related to exercise intensity and duration [7–9]. Exercise-induced elevations in cardiac troponin I (cTnI) and CK-MB are generally considered transient physiological responses following prolonged endurance exercise rather than indicators of irreversible myocardial injury [8,9]. However, substantial inter-individual variability exists in the magnitude and duration of these elevations, and the potential modulatory role of nutritional status remains incompletely understood. Although vitamin D has been suggested to exert cardioprotective effects, few studies have explored the effect of vitamin D_3_ supplementation on post-marathon cardiac biomarkers. Marathon running not only induces cardiac and skeletal muscle stress but also elicits transient alterations in immune cell distribution. Previous studies have documented post-marathon lymphocyte redistribution and changes in natural killer (NK) and natural killer T (NKT) cell frequencies during recovery, consistent with transient immune perturbations following prolonged endurance exercise [22,23]. These alterations are often discussed within the context of the “open-window” phenomenon following prolonged endurance exercise. However, it remains unclear how vitamin D status influences these immunophenotypic changes in marathon runners. Vitamin D plays a regulatory role in immune function, including modulation of T-cell differentiation and the phenotype and distribution of NK and NKT cells. Importantly, human studies conducted in the context of strenuous endurance exercise have reported that vitamin D₃ supplementation modulates exercise-induced immune perturbations and attenuates markers of muscle damage. These findings suggest that vitamin D status may influence post-exercise immune redistribution patterns in endurance athletes [24]. Therefore, assessing immune cell profiles is essential for understanding the broader protective effects of vitamin D in endurance athletes.

Despite extensive documentation of post-marathon biomarker elevations, it remains unclear whether nutritional interventions can meaningfully modify their magnitude or temporal dynamics. Moreover, cardiac, skeletal muscle, oxidative, and immune markers have rarely been examined concurrently within the same placebo-controlled intervention framework, and their coordinated recovery across the 0.5–24 h period remains insufficiently characterised. Accordingly, the present study was designed to determine whether vitamin D₃ supplementation modifies the integrated multi-system response to marathon running. We hypothesised that vitamin D₃ supplementation would attenuate post-marathon elevations in cardiac and skeletal muscle damage biomarkers, reduce oxidative stress responses and modulate antioxidant enzyme activity, and influence post-exercise immune cell redistribution patterns during the 0.5–24 h recovery period.

## 2. Materials and methods

### 2.1. Participants

In the present study, the sample size was calculated using the statistical power analysis software G*Power (version 3.1.9.7). For a two-way mixed-design ANOVA, with an effect size of 0.4, an *α* level of 0.05, and a power of 0.95, the required total sample size was determined to be 16 participants. Therefore, this research recruited 24 healthy amateur runners, aged 20–60 years, with no history of cardiovascular, liver, kidney, or immune diseases; diabetes; or acute exercise-related injuries. Only participants with serum 25(OH)D levels <30 ng/mL were enroled, in accordance with the Endocrine Society Clinical Practice Guideline, which defines serum 25(OH)D levels ≥30 ng/mL as sufficient for general health [25]. The participants were allocated to the vitamin D₃ and placebo groups using a matched allocation procedure. One participant from the vitamin D_3_ group and two from the placebo group withdrew during the race. Therefore, 21 participants were included in the final analysis. The characteristics of participants for the two groups are presented in Table 1.

**Table 1.** Participant characteristics.

Variable	Placebo group	Vitamin D_3_ group
Age (years)	44.00 ± 9.87	39.27 ± 7.93
Sex (male/female)	8/2	8/3
Height (cm)	168.10 ± 4.75	169.73 ± 6.31
Body weight (kg)	62.60 ± 7.78	63.95 ± 8.07
Body mass index (kg/m^2^)	22.12 ± 2.25	22.13 ± 1.79
Marathon finish time (min)	241.00 ± 44.33	220.09 ± 34.09

Data are presented as mean ± standard deviation (SD); Placebo group: *n* = 10, Vitamin D3 group: *n* = 11.

Before the commencement of this study, the participants received comprehensive information regarding the purpose, procedures, and other details of the experiment. After thoroughly reading and understanding the experimental instructions, the participants completed a health questionnaire and signed an informed consent form. For the 6 months leading up to this study, the participants were required to run at least 40 km per week. Furthermore, they were not allowed to participate in any other experiments or studies within 3 months of the present study and were instructed not to consume any nutritional supplements during the experimental period. Participants were advised to maintain their usual dietary habits, except for alcohol. This study was approved by the Institutional Review Board of Taipei Medical University (approval number: N202205045). This clinical trial was registered at ClinicalTrials.gov (registration ID: NCT05781620).

### 2.2. Experimental design

**Figure 1.**
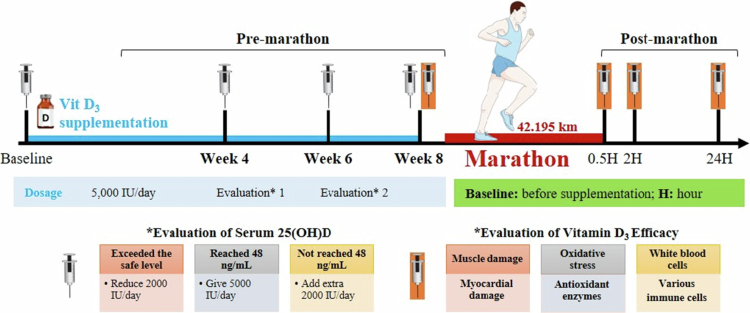
Experimental scheme.

For the present study, a single-blind, placebo-controlled, dose-titrated experimental design was adopted. Participants were allocated to either the vitamin D₃ group (*n* = 11) or the placebo group (*n* = 10) using a matched allocation procedure based on baseline serum 25-hydroxyvitamin D [25(OH)D] levels at Week 0. Participants were ranked by baseline 25(OH)D levels and sequentially assigned to groups to ensure comparable vitamin D status prior to supplementation. Personal-best marathon performance and average weekly running distance over the previous six months were recorded to confirm baseline equivalence but were not used for group assignment. Participants in the vitamin D₃ group received dose-titrated vitamin D₃ supplementation for 8 weeks leading up to a marathon race, whereas those in the placebo group received a matching placebo over the same period. Baseline blood samples were collected, and only participants with a serum 25(OH)D level <30 ng/mL were included. The serum 25(OH)D level was evaluated at 4 and 6 weeks after vitamin D₃ supplementation to monitor dose titration and ensure that the vitamin D₃ group attained a serum 25(OH)D level of ≥48 ng/mL after 8 weeks of supplementation. The target serum 25(OH)D level of ≥48 ng/mL was selected based on evidence suggesting that levels within the range of 40–60 ng/mL may confer additional extra-skeletal and performance-related benefits in athletes [26,27]. In our previous placebo-controlled intervention studies involving endurance-trained individuals, post-supplementation levels within this range (approximately 42–44 ng/mL) were associated with attenuation of exercise-induced oxidative stress and inflammatory responses [11,24]. Therefore, 48 ng/mL was selected as a mid-range target within the proposed optimal athletic range, while remaining well below levels associated with vitamin D toxicity. Subsequently, blood samples were collected 24 hours before the marathon (Pre), 0.5 h post-race (0.5H-post), 2 h post-race (2H-post), and 24 h post-race (24H-post) to measure myocardial biomarkers (CK-MB and cTnI), oxidative stress (TBARS, PC, superoxide dismutase [SOD], and catalase), and muscle damage (CK and lactate dehydrogenase [LDH]). The experimental procedure is depicted in Figure 1.

### 2.3. Supplementation

**Figure 2.**
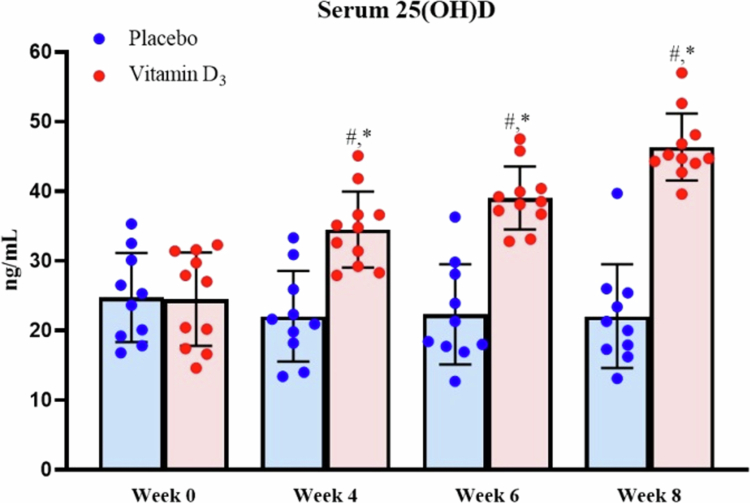
Serum 25(OH)D levels before and after 8-week supplementation. ^#^*p* < 0.05 compared with Week 0. *p < 0.05 compared with the placebo group.

The vitamin D_3_ group followed a dose titration regimen in which they consumed 5,000 IU of liquid-form vitamin D_3_ (Liquid Shield Vitamin D_3_ + E, Panion & BF Biotech, Taipei, Taiwan) daily after lunch for the first 4 weeks. Participants whose serum 25(OH)D level reached 48 ng/mL after this period continued with the same daily dosage. For participants whose serum 25(OH)D level did not reach 48 ng/mL, the dosage was increased by 2,000 IU. If the level exceeded the safe threshold, the dosage was reduced by 2,000 IU. The supplementation protocol for the 6th week mirrored that for the 4th week. In contrast, the placebo group consumed medium-chain triglycerides with the same colour, taste, and odour as the vitamin D_3_ supplement (Panion & BF Biotech). By the end of week 8, the serum 25(OH)D level was significantly higher in the vitamin D_3_ group than in the placebo group (Figure 2). To maintain participant blinding despite the dose-titration protocol, the placebo group underwent a parallel mock dose-adjustment procedure at the same predefined time points. Although the placebo solution contained only medium-chain triglycerides, the administered volume (number of drops) was adjusted according to the same schedule as the vitamin D₃ group to ensure identical dosing procedures and appearance between groups. Interim serum 25(OH)D levels were reviewed by a designated research assistant responsible for supplementation management, who was not involved in outcome assessment or statistical analysis. Laboratory personnel conducting biochemical assays and investigators performing statistical analyses were blinded to group allocation throughout the study.

### 2.4. Marathon details and post-race blood collection

In this study, the marathon distance was 42.195 km; the participants completed a full marathon. Participants completed one of two official marathon events. The proportion of vitamin D₃ and placebo participants was comparable across the two races to minimise potential race-specific clustering effects. The study was conducted under real-world marathon conditions; therefore, in-race hydration and fuelling strategies were not experimentally standardised or formally recorded. However, participants were instructed not to consume any additional nutritional supplements during the intervention period, as specified in the IRB-approved inclusion criteria. As mentioned, blood samples (total volume/participant, 31 mL) were collected at seven time points: before supplementation (1 mL); 4 (1 mL) and 6 (1 mL) weeks after supplementation; 24 h before the race (7 mL); and 0.5 (7 mL), 2 (7 mL), and 24 h (7 mL) after the race. These samples were analysed for cardiac biomarkers (CK-MB and cTnI), oxidative stress markers (TBARS, PC, SOD, and catalase), and muscle damage markers (CK and LDH) to evaluate the effects of vitamin D_3_ supplementation.

### 2.5. Blood sampling and analysis

Blood samples were collected at multiple predefined time points depending on the outcome measure. For assessment of vitamin D status, venous blood samples (1 mL) were obtained prior to supplementation (Week 0) and at Weeks 4, 6, and 8 during the supplementation period. Serum 25(OH)D levels were measured using a fully automated immunoassay analyser (Cobas e801; Roche Diagnostics, Mannheim, Germany). To evaluate marathon-induced physiological responses, blood samples were collected at predefined time points (Pre, 0.5H-post, 2H-post, and 24H-post). The pre-sample was collected 24 h before the marathon at a standardised time of day. Participants were instructed to avoid strenuous exercise and alcohol consumption for at least 24 h prior to sampling. These samples were analysed to determine biomarkers of cardiac injury, oxidative stress, muscle damage, and immune cell responses. All blood samples were allowed to clot at room temperature for 30 min and then centrifuged at 3,000 rpm for 10 min at 4 °C (5702 R; Eppendorf, Hamburg, Germany). The resultant serum was aliquoted and stored at −80 °C until analysis.

#### 2.5.1. Biochemical analysis

The cardiac biomarkers CK-MB (ng/mL) and cTnI (pg/mL) were measured using an automated immunoassay analyser (Beckman Coulter DxI 800, Brea, CA, USA) according to the manufacturer’s instructions. The detection limit for CK-MB was 6 ng/mL according to the manufacturer’s specifications. A cTnI level > 40 pg/mL was considered positive, corresponding to the assay-specific 99th percentile upper reference limit for healthy individuals.

TBARS is a biomarker of lipid oxidation damage. The aforementioned samples were thawed at room temperature, and the TBARS reagent was preheated to eliminate any precipitates. Then, 1,000 μL of this reagent was mixed with 200 μL of standard solution or serum sample in a 4-mL polypropylene tube. This mixture was heated and then placed on ice to stop the reaction. Subsequently, it was centrifuged (5702 R) to collect the supernatant. Absorbance was measured at 535 nm by using 200 μL of the supernatant. The level of TBARS (μM) was determined using a standard curve.

PC is a biomarker of protein oxidation damage [28,29]. The serum samples of this study were diluted and added to a microplate, which was incubated at 4 °C for 16 h. The next day, the microplate was washed and incubated (in the dark, at room temperature) for 45 min with 2,4-dinitrophenylhydrazine solution. After incubation, the microplate was washed, blocked, and incubated with antibodies. Another washing step followed this. Finally, the substrate solution was added. Absorbance was measured at 450 nm. The PC level (nmol/mg) was determined using a regression equation.

SOD (U/mL) was measured by adding water-soluble tetrazolium solution to the microplates containing the serum samples. After adding the enzyme solution, the microplates were incubated at 37 °C for 20 min. Absorbance was measured at 450 nm. The level of SOD was determined using a standard curve.

Catalase (nmol/min/mL) was measured using Cayman’s catalase assay kit. Blood serum or standard solution was mixed with 1X assay buffer and methanol. After, hydrogen peroxide was added to stop the reaction. Next, the Purpald reagent was added for analysis. Absorbance was measured at 540 nm, and catalase level was determined from a standard curve.

The muscle damage markers CK (U/L) and LDH (U/L) were measured using an automated immunoassay analyser (Beckman AU5820; Ireland).

#### 2.5.2. Isolation of peripheral blood mononuclear cells

Whole blood was purified using heparin tubes. Peripheral blood mononuclear cells (PBMCs) were isolated through Ficoll–Hypaque density gradient centrifugation and were stored as frozen pellets at −80 °C until further use.

#### 2.5.3. Immunoprofiling

The Attune NxT flow cytometry software (Invitrogen) was used to analyse PBMCs stained with a 10-colour antibody panel. Detailed gating was performed using previously described protocols [30]. The following immune cell subsets were predefined and analysed: naïve total T helper (Th) cells, CD56^bright NKT cells (CD56b NKT), CD56^dim NKT cells (CD56d NKT), CD56^bright NK cells (CD56b NK), active total NK cells, total B cells, and active total Th cells.

### 2.6. Statistical analysis

Statistical analyses were performed using IBM SPSS Statistics for Windows, version 22.0 (IBM Corp., Armonk, NY, USA). Normality of distribution was assessed using the Shapiro–Wilk test. Continuous biochemical markers and selected haematological variables (cardiac biomarkers, muscle damage markers, oxidative stress markers, and antioxidant enzymes) were analysed on the natural log-transformed scale prior to inferential testing to improve distributional characteristics and variance homogeneity. For white blood cell parameters and immune cell subsets, statistical analyses were performed on the original scale, as these data demonstrated acceptable distributional characteristics and met the assumptions for parametric testing without the need for logarithmic transformation. A two-way mixed-design analysis of variance (ANOVA) was conducted for each dependent variable, with group (vitamin D₃ vs. placebo) as the between-subject factor and time (Pre, 0.5H-post, 2H-post, and 24H-post) as the within-subject factor. Main effects of group and time, as well as the group × time interaction, were evaluated. Mauchly’s test of sphericity was performed, and Greenhouse–Geisser corrections were applied when the assumption of sphericity was violated. In accordance with statistical conventions, simple main effects were examined only when a significant group × time interaction was observed. Bonferroni-adjusted post hoc comparisons were applied; for comparisons across the three post-race time points, the adjusted significance threshold was set at *p* < 0.0167 (0.05/3). When no significant interaction was detected, interpretation focused on the main effects. Effect sizes were reported as partial eta squared (partial *η*²) for ANOVA results. Cohen’s d was calculated for pairwise comparisons where applicable. Primary outcomes were predefined as cardiac and muscle damage biomarkers (CK, CK-MB, cTnI, and LDH). Secondary outcomes included oxidative stress markers, antioxidant enzymes, white blood cell parameters, and immune cell subsets. Correlation analyses were performed using Pearson’s correlation coefficients. Linearity and normality assumptions were visually inspected using scatterplots. Pearson correlation analyses were conducted to explore potential associations between immune cell redistribution and biochemical stress markers during post-marathon recovery. Correlation analyses were conducted for exploratory purposes and were not pre-specified as confirmatory endpoints. Given the number of tested immune subsets, biochemical markers, and time points, no formal multiple-comparison adjustment was applied. Accordingly, these findings should be interpreted cautiously and considered hypothesis-generating rather than confirmatory. Data are presented as mean ± standard deviation (SD). For variables analysed on the natural log-transformed scale, figures are presented using raw values for descriptive purposes, whereas statistical inferences were based on analyses performed on the log-transformed data. Statistical significance was set at *α* = 0.05 unless otherwise specified.

## 3. Results

Serum 25(OH)D levels were analysed using a two-way mixed-design ANOVA with group (vitamin D₃ vs. placebo) as the between-subject factor and time (Week 0, Week 4, Week 6, and Week 8) as the within-subject factor. A significant group × time interaction was observed (*F*(1.99, 37.81) = 54.19, *p* < 0.001, partial *η*² = .74), indicating that changes in serum 25(OH)D over the supplementation period differed between groups. Significant main effects of group (*F*(1, 19) = 30.44, *p* < .001, partial *η*² = .62) and time (*F*(1.99, 37.81) = 33.41, *p* < 0.001, partial *η*² = 0.64) were also detected. Bonferroni-adjusted simple main effects analyses revealed that serum 25(OH)D levels were significantly higher in the vitamin D₃ group compared with the placebo group at Weeks 4, 6, and 8. Furthermore, serum 25(OH)D levels increased significantly from Week 0 at Weeks 4, 6, and 8 in the vitamin D₃ group, whereas no significant changes were observed in the placebo group (Figure 2).

### 3.1. Effects of vitamin D_3_ supplementation on muscle and myocardial damage

**Figure 3.**
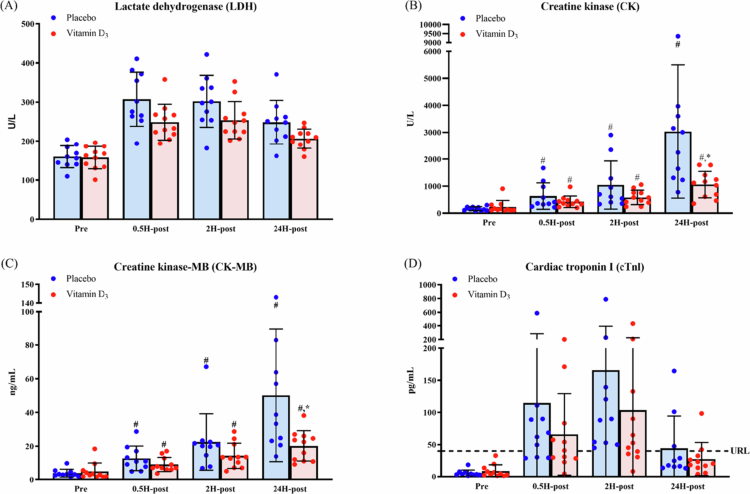
Changes in (A) lactate dehydrogenase, (B) creatine kinase, (C) creatine kinase-MB, and (D) cardiac troponin I levels after a marathon race. ^#^*p* < 0.0167 compared with Pre. *p < 0.0167 compared with the placebo group. In panel (D), the dashed horizontal line indicates the assay-specific upper reference limit (URL) for cTnI (40 pg/mL). Abbreviations: Pre, 24 h before the marathon; 0.5H-post, 0.5 h post-race; 2H-post, 2 h post-race; 24H-post, 24 h post-race. Note: Data are presented as raw mean ± SD. Statistical analyses were performed on natural log-transformed data.

As shown in Table 2 and Figure 3, significant group × time interactions were observed for CK and CK-MB. Bonferroni-adjusted simple main effects analyses indicated that CK and CK-MB levels were significantly higher at 0.5H-post, 2H-post, and 24H-post than at Pre in both groups; however, levels of both biomarkers were significantly lower in the vitamin D₃ group than in the placebo group at 24H-post. No significant group × time interactions were found for LDH or cTnI. A significant main effect of time was observed for both variables. Bonferroni-adjusted post hoc analyses further revealed that LDH and cTnI levels were significantly higher at 0.5H-post, 2H-post, and 24H-post than at Pre. As a descriptive observation, no participants tested positive for cTnI before the race, and post-race cTnI positivity rates were numerically lower in the vitamin D₃ group at 0.5H-post, 2H-post, and 24H-post (45.45% vs. 70%, 63.64% vs. 100%, and 18.18% vs. 30%, respectively).

**Table 2.** Results of a two-way mixed-design ANOVA examining the effects of group (vitamin D₃ vs. placebo) and time (Pre, 0.5H-post, 2H-post, and 24H-post) on muscle and myocardial damage biomarkers.

Variable	Effect	*F*(df1, df2)	*p*	partial *η*²
LDH	Group	*F*(1, 19) = 3.99	0.06	0.17
	Time	*F*(1.51, 28.66) = 84.40	<0.01	0.82
	Group × Time	*F*(1.51, 28.66) = 2.18	0.14	0.10
CK	Group	*F*(1, 19) = 2.72	0.12	0.13
	Time	*F*(2.08, 39.49) = 119.82	<0.01	0.86
	Group × Time	*F*(2.08, 39.49) = 6.63	<0.01	0.26
CK-MB	Group	*F*(1, 19) = 2.74	0.11	0.13
	Time	*F*(2.09, 39.65) = 106.19	<0.01	0.85
	Group × Time	*F*(2.09, 39.65) = 3.99	0.03	0.17
cTnI	Group	*F*(1, 16) = 0.02	0.90	<0.01
	Time	*F*(1.73, 27.75) = 57.25	<0.01	0.78
	Group × Time	*F*(1.73, 27.75) = 0.68	0.50	0.04

Abbreviations: LDH, lactate dehydrogenase; CK, creatine kinase; CK-MB, creatine kinase–MB; cTnI, cardiac troponin I; Pre, 24 h before the marathon; 0.5H-post, 0.5 h post-race; 2H-post, 2 h post-race; 24H-post, 24 h post-race.

### 3.2. Effects of vitamin D_3_ supplementation on oxidative stress and antioxidant enzymes

**Figure 4.**
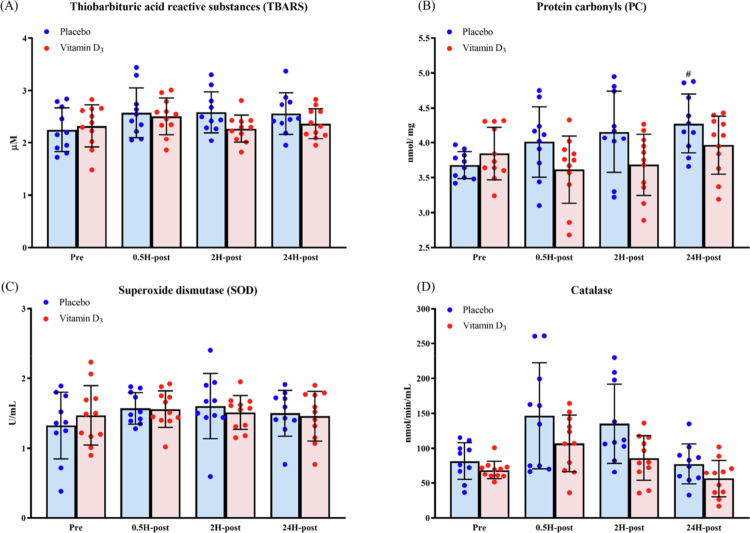
Changes in (A) thiobarbituric acid reactive substances, (B) protein carbonyls, (C) superoxide dismutase, and (D) catalase levels after a marathon race. ^#^*p* < 0.0167 compared with Pre. *p < 0.0167 compared with the placebo group. Abbreviations: Pre, 24 h before the marathon; 0.5H-post, 0.5 h post-race; 2H-post, 2 h post-race; 24H-post, 24 h post-race. Note: Data are presented as raw mean ± SD. Statistical analyses were performed on natural log-transformed data.

As shown in Table 3 and Figure 4, a significant group × time interaction was observed for PC. Bonferroni-adjusted simple main effects analyses indicated that PC levels were significantly higher at 24H-post than at Pre in the placebo group, whereas no significant changes were observed in the vitamin D₃ group. No significant between-group differences were detected at any individual time point. No significant group × time interactions were observed for TBARS, SOD, or catalase. For TBARS and catalase, significant main effects of time were detected. Bonferroni-adjusted post hoc analyses revealed that TBARS levels were significantly higher at 0.5H-post than at Pre, whereas catalase levels were significantly higher at 0.5H-post and 2H-post than at Pre. For SOD, neither the main effect of group nor time was statistically significant.

**Table 3.** Results of a two-way mixed-design ANOVA examining the effects of group (vitamin D₃ vs. placebo) and time (Pre, 0.5H-post, 2H-post, and 24H-post) on oxidative stress and antioxidant enzyme biomarkers.

Variable	Effect	*F*(df1, df2)	*p*	partial *η*²
TBARS	Group	*F*(1, 19) = 0.72	0.41	0.04
	Time	*F*(3, 57) = 4.46	0.01	0.19
	Group × Time	*F*(3, 57) = 2.23	0.09	0.11
PC	Group	*F*(1, 19) = 2.93	0.10	0.13
	Time	*F*(3, 57) = 4.19	0.01	0.18
	Group × Time	*F*(3, 57) = 3.44	0.02	0.15
SOD	Group	*F*(1, 19) = 0.05	0.82	<0.01
	Time	*F*(3, 57) = 2.20	0.10	0.10
	Group × Time	*F*(3, 57) = 0.79	0.50	0.04
Catalase	Group	*F*(1, 19) = 3.60	0.07	0.16
	Time	*F*(3, 57) = 25.24	<0.01	0.57
	Group × Time	*F*(3, 57) = 1.50	0.22	0.07

Abbreviations: TBARS, thiobarbituric acid reactive substances; PC, protein carbonyls; SOD, superoxide dismutase; Pre, 24 h before the marathon; 0.5H-post, 0.5 h post-race; 2H-post, 2 h post-race; 24H-post, 24 h post-race.

### 3.3. Effects of vitamin D_3_ supplementation on white blood cells and lymphocytes

**Figure 5.**
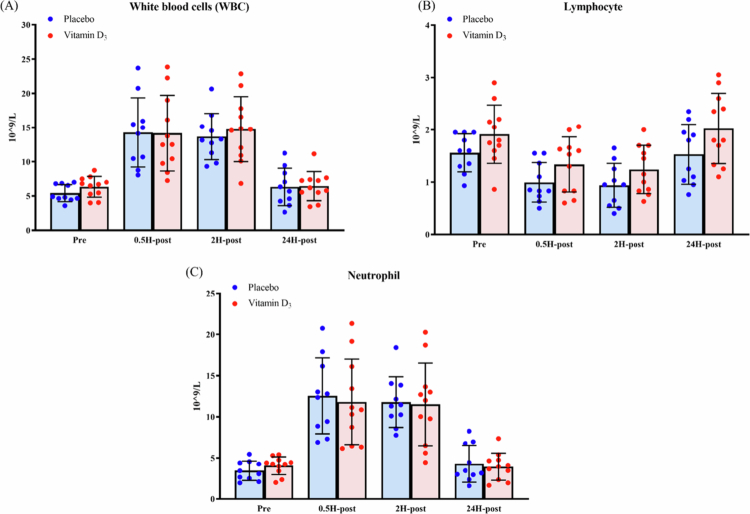
Changes in (A) white blood cell counts, (B) lymphocyte counts, and (C) neutrophil counts after a marathon race. Abbreviations: Pre, 24 h before the marathon; 0.5H-post, 0.5 h post-race; 2H-post, 2 h post-race; 24H-post, 24 h post-race.

As shown in Table 4 and Figure 5, no significant group × time interactions were observed for WBC, lymphocyte, or neutrophil. Significant main effects of time were observed for all three variables. Bonferroni-adjusted post hoc analyses revealed that WBC and neutrophil counts were significantly higher at 0.5H-post and 2H-post than at Pre, whereas lymphocyte counts were significantly lower at 0.5H-post and 2H-post than at Pre.

**Table 4.** Results of a two-way mixed-design ANOVA examining the effects of group (vitamin D₃ vs. placebo) and time (Pre, 0.5H-post, 2H-post, and 24H-post) on white blood cells and lymphocytes.

Variable	Effect	*F* (df1, df2)	*p*	partial *η*²
WBC	Group	*F*(1, 19) = 0.16	0.70	0.01
	Time	*F*(1.50, 28.49) = 71.49	<0.01	0.79
	Group × Time	*F*(1.50, 28.49) = 0.29	0.69	0.02
Lymphocyte	Group	*F*(1, 19) = 3.86	0.06	0.17
	Time	*F*(3, 57) = 31.16	<0.01	0.62
	Group × Time	*F*(3, 57) = 0.42	0.70	0.02
Neutrophil	Group	*F*(1, 19) = 0.03	0.87	<0.01
	Time	*F*(1.65, 31.34) = 65.61	<0.01	0.78
	Group × Time	*F*(1.65, 31.34) = 0.25	0.74	0.01

Abbreviations: WBC, white blood cells; Pre, 24 h before the marathon; 0.5H-post, 0.5 h post-race; 2H-post, 2 h post-race; 24H-post, 24 h post-race.

### 3.4. Effects of vitamin D_3_ supplementation on various immune cell*s*

**Figure 6.**
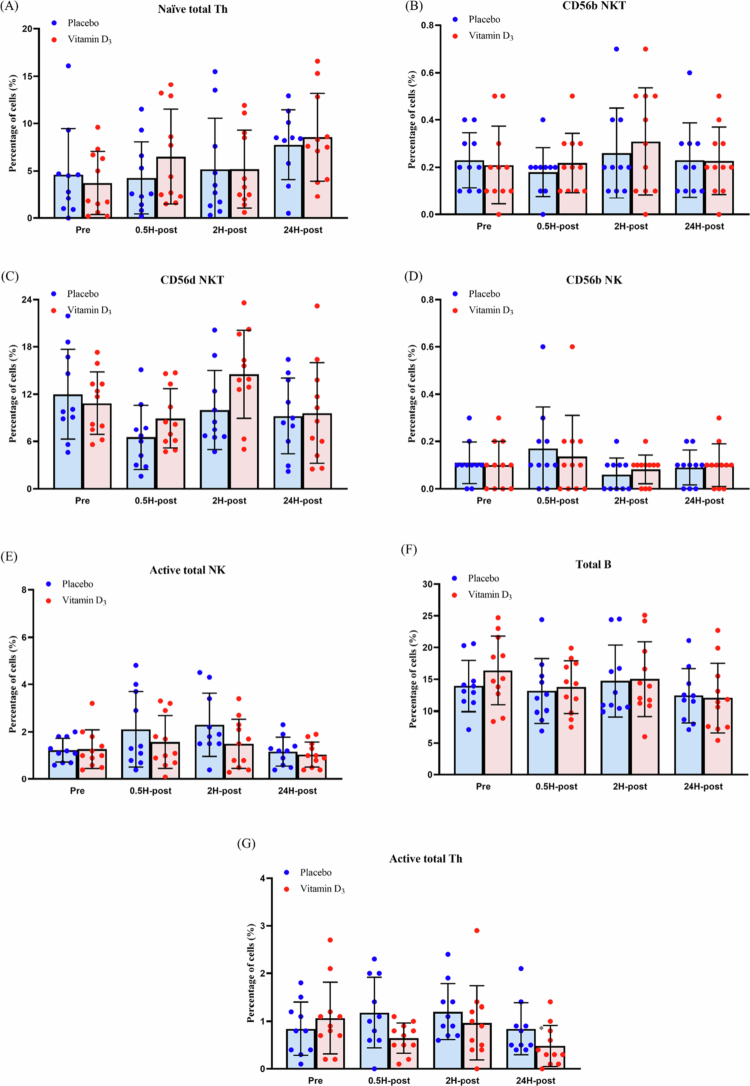
Changes in the percentages of (A) Naïve total Th, (B) CD56b NKT, (C) CD56d NKT, (D) CD56b NK, (E) Active total NK, (F) Total B, and (G) Active total Th. Abbreviations: Th, T helper cells; NK, natural killer cells; NKT, natural killer T cells; B, B cells; CD56b, CD56^bright; CD56d, CD56^dim; Active, CD69^+^; Pre, 24 h before the marathon; 0.5H-post, 0.5 h post-race; 2H-post, 2 h post-race; 24H-post, 24 h post-race.

As shown in Table 5 and Figure 6, no significant group × time interactions were observed for Naïve total Th, CD56b NKT, CD56d NKT, CD56b NK, Active total NK, Total B, or Active total Th. Significant main effects of time were observed for selected immune cell subsets. Bonferroni-adjusted post hoc analyses revealed that Naïve total Th percentages were significantly higher at 24H-post than at Pre, whereas Total B percentages were significantly lower at 24H-post than at Pre. Active total NK percentages were significantly higher at 2H-post than at Pre. Time effect was also observed for CD56d NKT; however, no significant pairwise differences were identified relative to Pre after Bonferroni adjustment.

**Table 5.** Results of a two-way mixed-design ANOVA examining the effects of group (vitamin D₃ vs. placebo) and time (Pre, 0.5H-post, 2H-post, and 24H-post) on immune cell populations.

Variable	Effect	*F* (df1, df2)	*p*	partial *η*²
Naïve Total Th	Group	*F*(1, 19) = 0.14	0.71	0.01
	Time	*F*(3, 57) = 5.56	<0.01	0.23
	Group × Time	*F*(3, 57) = 0.83	0.48	0.04
CD56b NKT	Group	*F*(1, 19) = 0.08	0.78	<0.01
	Time	*F*(2.26, 42.87) = 2.63	0.08	0.12
	Group × Time	*F*(2.26, 42.87) = 0.54	0.61	0.03
CD56d NKT	Group	*F*(1, 19) = 1.46	0.24	0.07
	Time	*F*(2.04, 38.80) = 3.98	0.03	0.17
	Group × Time	*F*(2.04, 38.80) = 1.46	0.24	0.07
CD56b NK	Group	*F*(1, 19) = 0.01	0.93	<0.01
	Time	*F*(1.94, 36.90) = 3.06	0.06	0.14
	Group × Time	*F*(1.94, 36.90) = 0.38	0.68	0.02
Active total NK	Group	*F*(1, 19) = 0.96	0.34	0.05
	Time	*F*(1.79, 34.05) = 7.87	<0.01	0.29
	Group × Time	*F*(1.79, 34.05) = 1.78	0.19	0.09
Total B	Group	*F*(1, 19) = 0.14	0.71	0.01
	Time	*F*(1.94, 36.89) = 5.11	0.01	0.21
	Group × Time	*F*(1.94, 36.89) = 1.00	0.38	0.05
Active total Th	Group	*F*(1, 19) = 1.57	0.23	0.08
	Time	*F*(3, 57) = 2.48	0.07	0.12
	Group × Time	*F*(3, 57) = 2.10	0.11	0.10

Abbreviations: Th, T helper cells; NK, natural killer cells; NKT, natural killer T cells; B, B cells; CD56b, CD56^bright; CD56d, CD56^dim; Active, CD69+; Pre, 24 h before the marathon; 0.5H-post, 0.5 h post-race; 2H-post, 2 h post-race; 24H-post, 24 h post-race.

**Figure 7.**
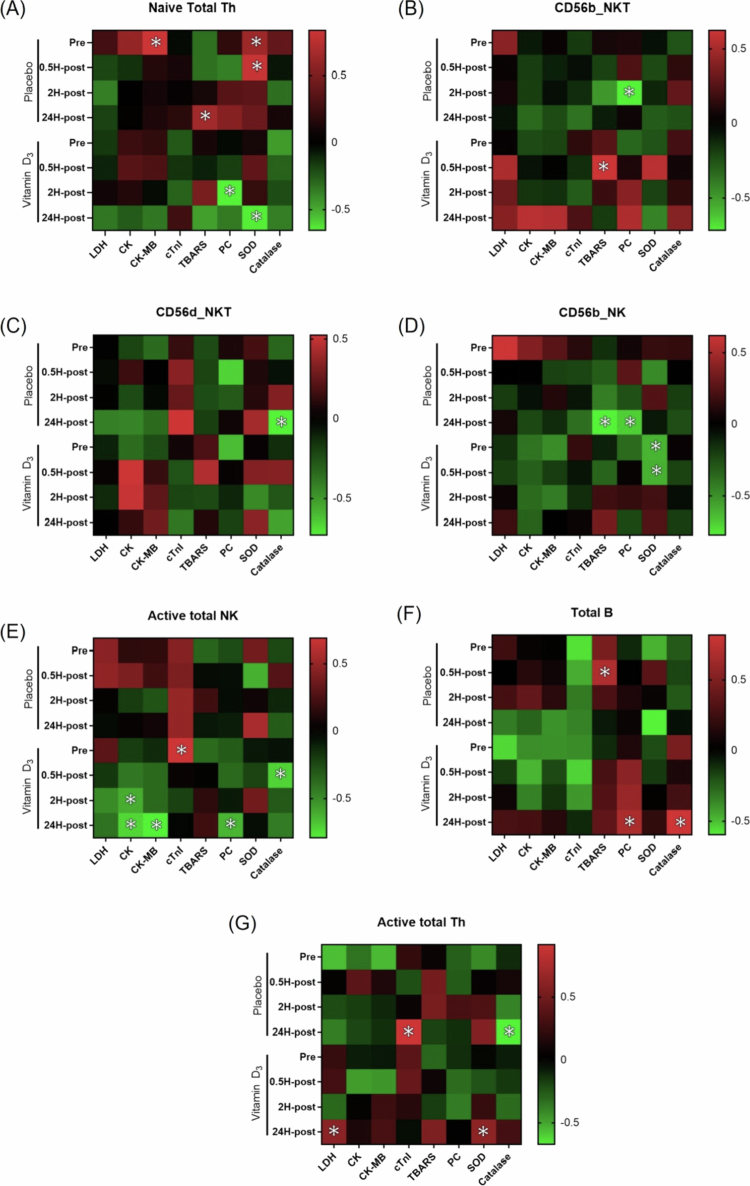
Heatmap of Pearson correlation coefficients between biochemical markers and immune cell populations at Pre, 0.5H-post, 2H-post, and 24H-post. Colour intensity represents the strength and direction of the correlation coefficient (*r*). *p < 0.05 indicates statistical significance. Abbreviations: CK, creatine kinase; CK-MB, creatine kinase-MB; cTnI, cardiac troponin I; LDH, lactate dehydrogenase; Th, T helper cells; NK, natural killer cells; NKT, natural killer T cells; B, B cells; CD56b, CD56^bright; CD56d, CD56^dim; Active, CD69^+^; Pre, 24 h before the marathon; 0.5H-post, 0.5 h post-race; 2H-post, 2 h post-race; 24H-post, 24 h post-race.

Correlation analyses between clinical biomarkers (LDH, CK, CK-MB, cTnI, TBARS, PC, SOD, and catalase) and immune cell populations were performed at pre- and post-race time points within each group (Figure 7). In the placebo group, significant associations were primarily observed between immune cell subsets and markers of oxidative stress and muscle damage at isolated time points. For example, Naïve total Th cells were positively correlated with CK-MB and PC at Pre, whereas CD56d NKT cells were correlated with catalase at 24H-post. Total B cells showed an association with TBARS shortly after the race. In the vitamin D₃ group, time-specific associations were also observed during the recovery phase. Naïve total Th cells were correlated with PC at 2H-post, and CD56b NKT cells were associated with TBARS immediately after the race. CD56b NK cells were correlated with both PC and SOD at 2H-post. Correlations involving Active total NK and Total B cells were observed at 24H-post, including associations with CK, catalase, SOD, and LDH.

## 4. Discussion

Marathon running imposes substantial multi-system physiological stress, including skeletal muscle disruption, transient cardiac biomarker release, oxidative stress, and immune cell redistribution [5,6,31,32]. In the present study, the overall post-race response was characterised by increases in muscle damage, cardiac-related, and oxidative stress biomarkers, together with time-dependent alterations in leucocyte and immune cell profiles. Within this broader response pattern, vitamin D₃ supplementation was associated with differential temporal responses in selected variables, particularly CK, CK-MB, and PC, whereas most immune-related outcomes appeared to reflect the general recovery process after marathon running, with only modest descriptive differences between groups. These findings suggest that the physiological effects of marathon running are systemic and dynamic, and that vitamin D₃ status may be associated with modulation of selected aspects of post-exercise recovery rather than a uniform effect across all measured systems.

In addition to elevations in muscle and cardiac biomarkers, oxidative stress may represent a central mechanism linking post-marathon physiological responses. Among the oxidative stress markers evaluated, protein oxidation appeared to show the clearest group-related divergence during recovery, whereas lipid peroxidation and enzymatic antioxidant responses were more consistent with a generalised exercise-related response across both groups. Previous marathon and ultra-endurance studies have reported elevations in lipid and protein oxidation during recovery [6], along with increased muscle and cardiac biomarkers [10]. However, in contrast to laboratory cycling studies demonstrating vitamin D–associated attenuation of oxidative stress [17,18], we did not observe between-group differences in TBARS during early recovery. This divergence may reflect differences in exercise modality and oxidative kinetics. Marathon running involves prolonged mechanical loading and eccentric stress, which have been linked to structural muscle damage and oxidative protein modification [6]. Lipid peroxidation and protein oxidation represent distinct biochemical pathways of oxidative damage. Protein carbonyl formation has been described as an early and relatively stable marker of oxidative protein modification [28,29], whereas lipid peroxidation products, such as TBARS, may reflect membrane lipid oxidation processes that vary with exercise intensity and recovery dynamics [6]. Training status, baseline vitamin D deficiency, and real-world field conditions may further influence oxidative responses.

Protein carbonyl formation reflects irreversible oxidative modification of structural and membrane-associated proteins [28,29]. Exercise-induced release of cytosolic enzymes such as CK and CK-MB may be related, at least in part, to transient alterations in membrane permeability [9]. The parallel temporal patterns observed for PC and selected biomarkers may reflect shared responses to exercise-induced oxidative and membrane stress. Although causality cannot be established, the proposed redox–membrane framework may represent one potential explanation for the differential recovery patterns observed in selected biomarkers, consistent with prior reports that vitamin D supplementation may selectively modulate exercise-induced oxidative stress and antioxidant responses [17,18]. The overall pattern of SOD and catalase responses suggests that enzymatic antioxidant activity was influenced more strongly by the acute exercise challenge than by vitamin D₃ status during prolonged endurance stress. This pattern may indicate that protein oxidation pathways are more sensitive to vitamin D status than endogenous enzymatic systems. In addition to laboratory-based endurance protocols, recent ultramarathon field studies have reported that vitamin D supplementation may attenuate inflammation and modulate muscle and cardiac biomarkers following extreme endurance events [33,34], although effects on oxidative stress markers remain largely unexplored.

Elevations in cardiac biomarker levels have been reported in both pathological conditions involving impaired blood supply, such as myocardial infarction, and in response to strenuous exercise [35]. In the present study, substantial post-race increases in CK-MB and cTnI were observed, indicating marked cardiac-related physiological stress after marathon running. However, post-exercise troponin elevations in healthy endurance athletes are generally transient and are commonly regarded as physiological rather than indicative of overt myocardial injury [8]. Within this interpretive framework, the lower cTnI positivity rates observed in the vitamin D₃ group may be consistent with attenuated transient myocardial stress during extreme endurance loading. In our study, cTnI returned toward baseline within 24 hours without clinical evidence of acute coronary syndrome, supporting the interpretation of reversible exercise-related myocardial stress. In contrast, CK-MB appeared to show a more distinct divergence in recovery pattern between groups, suggesting that vitamin D₃ status may be associated with modulation of this response after prolonged endurance exercise. In this context, attenuation should be interpreted as a reduction in the magnitude or duration of exercise-related biomarker elevations within the physiological range typically observed in healthy endurance athletes, rather than as evidence of overt myocardial injury. Mechanistic explanations involving transient membrane permeability remain speculative, as myocardial structure and membrane integrity were not directly assessed. Exercise-induced elevations in cardiac biomarkers have previously been described as transient and potentially leading to false-positive interpretations of myocardial infarction [36]. Long-term participation in extreme endurance exercise may be associated with structural remodelling; however, athletes generally demonstrate preserved functional capacity and no clear evidence of increased mortality risk [31,37]. Collectively, these findings are consistent with the hypothesis that sufficient vitamin D status may modulate exercise-related biomarker responses; however, the causal mechanisms remain to be clarified.

Vitamin D exerts immunomodulatory effects through binding to the vitamin D receptor (VDR), which is expressed in multiple immune cell types, including monocytes and T lymphocytes [38–42]. Activation of VDR signalling has been associated with modulation of cytokine production and NK/T cell–related immune responses, promoting a more regulatory immune profile [43–45]. However, the present study assessed only circulating 25(OH)D levels and did not directly evaluate downstream VDR-mediated pathways. The immune cell data suggested broadly time-dependent redistribution after marathon running, with descriptive differences in recovery dynamics between groups, consistent with previously reported exercise-induced immune redistribution [22,23]. The vitamin D₃ group appeared to show relatively preserved proportions of naïve Th and NKT cells during early recovery, whereas the placebo group appeared to show greater late-phase increases in B cells and activated Th subsets. These patterns may reflect variation in immune redistribution kinetics under differing vitamin D status. However, these observations were modest and descriptive in nature, and therefore should be interpreted cautiously. Considering the multiplicity of tested associations across immune subsets, biomarkers, and time points, these correlations should be regarded as exploratory signals rather than evidence of causal relationships.

Epidemiological evidence linking vitamin D deficiency to several autoimmune diseases [46] underscores the immunomodulatory role of vitamin D, particularly in maintaining balanced immune regulation, by promoting immune tolerance and mitigating excessive inflammation [47,48]. The present study provides additional insight into the potential effects of vitamin D status in individuals who regularly engage in marathon running or other forms of intensive endurance training. However, this study has several limitations, including a relatively small sample size; limited dietary control apart from vitamin D supplementation during the intervention period; the absence of strict standardisation or formal recording of in-race behaviours (e.g. hydration, fuelling strategies, caffeine intake, and NSAID use); and the lack of mechanistic investigations into vitamin D–mediated immune modulation in marathon runners. In addition, haemoglobin and haematocrit were not measured at post-race time points; therefore, plasma volume correction for potential hemoconcentration was not performed. Furthermore, only participants with baseline serum 25(OH)D < 30 ng/mL were included, and supplementation targeted levels ≥48 ng/mL. This design limits generalisability to vitamin D–replete individuals and does not allow inference regarding dose–response relationships. Accordingly, further research is warranted to expand our understanding of vitamin D–mediated immunomodulation mechanisms across diverse physiological contexts. Importantly, these immunological alterations should be interpreted within the broader context of multi-systemic physiological responses to prolonged endurance exercise, while the potential role of vitamin D in post-marathon recovery warrants further investigation.

## 5. Conclusion

In the present study, vitamin D₃ supplementation was associated with increased serum 25(OH)D levels and differential temporal responses in CK, CK-MB, and PC, with lower 24-hour post-race elevations observed in the vitamin D₃ group. In addition, immune cell subsets showed broadly time-dependent recovery patterns, with modest descriptive differences between groups. These patterns may be consistent with a potential influence of vitamin D₃ status on post-exercise redistribution dynamics of lymphocytes and NKT/NK cells. Collectively, these findings suggest that vitamin D₃ status may be associated with differential physiological responses to prolonged endurance exercise, whereas immune alterations were primarily time-dependent and exploratory. Further studies with larger sample sizes are warranted to clarify the underlying mechanisms and to determine the clinical relevance of these observations.

## Data Availability

All relevant data are included in the article and its supplementary materials.
